# Time Trends of Etiology, Treatment, and Long-Term Outcomes Among Patients with Left Ventricular Thrombus

**DOI:** 10.31083/j.rcm2410298

**Published:** 2023-10-20

**Authors:** Boqun Shi, Yanjun Song, Lie Ma, Xieraili Tiemuerniyazi, Jinpeng Liu, Rui Zhang, Chenxi Song, Lei Jia, Dong Yin, Hongjian Wang, Wei Feng, Weihua Song, Ke-Fei Dou

**Affiliations:** ^1^Cardiometabolic Medicine Center, Department of Cardiology, Fuwai Hospital, Chinese Academy of Medical Sciences & Peking Union Medical College/National Center for Cardiovascular Diseases, 100037 Beijing, China; ^2^Coronary Heart Disease Center, Department of Cardiology, Fuwai Hospital, Chinese Academy of Medical Sciences & Peking Union Medical College/National Center for Cardiovascular Diseases, 100037 Beijing, China; ^3^State Key Laboratory of Cardiovascular Disease, 102308 Beijing, China; ^4^Department of Cardiovascular Surgery, Fuwai Hospital, Chinese Academy of Medical Sciences & Peking Union Medical College/National Center for Cardiovascular Diseases, 100037 Beijing, China; ^5^National Clinical Research Center for Cardiovascular Diseases, 100037 Beijing, China

**Keywords:** left ventricular thrombosis, anticoagulation, direct oral anticoagulants, vitamin K antagonists

## Abstract

**Background:**

Recommendations for drug treatment of left ventricular thrombus (LVT) are based on the ST-segment elevation myocardial infarction (STEMI) guidelines; however, the etiology of LVT has changed. Due to the lack of evidence regarding LVT treatment in the heart failure population, current heart failure guidelines do not cover LVT treatment. We sought to review the etiology of LVT and changes in antithrombotic therapy over the previous 12 years and explore the impact of anticoagulation treatment from a single center’s experience.

**Methods:**

From January 2009 to June 2021, we studied 1675 patients with a discharge diagnosis of LVT at a single center to investigate the clinical characteristics, incidence of all-cause death, cardiovascular death, ischemic stroke, major adverse cardiac and cerebrovascular events (MACCE), systemic embolism (SE), and major bleeding events. Patients were divided into an anticoagulant group and a non-anticoagulant group according to whether they received oral anticoagulant therapy at discharge.

**Results:**

The study included 909 patients (anticoagulation, 510; no anticoagulation, 399). While overall antiplatelet therapy dramatically decreased, more patients with LVT received oral anticoagulation in 2021 (74.0%) than in 2009 (29.6%). In addition, more than half of the patients had heart failure with reduced ejection fraction (HFrEF) each year. The all-cause mortality was 17.3% during 3.8 years of follow-up. The incidences of cardiovascular death, stroke, MACCE, SE, and major bleeding were 16.0%, 3.3%, 19.8%, 5.1%, and 1.7%, respectively. The anticoagulation group had a significantly higher proportion of dilated cardiomyopathy than the non-anticoagulation group (24.7% vs. 5.5%, *p* < 0.001), and a lower LVEF (34.0 vs. 41.0, *p* < 0.001). The anticoagulation group also had a higher probability of adverse events on long-term follow-up (*p* > 0.05). A multivariable competing risk regression model found no significant difference in all six endpoints between the groups (all *p* > 0.05). Similar results were found by matched and weighted data analysis. Diabetes mellitus (hazard ratio (HR), 1.42; 95% confidence interval (CI), 1.04–1.93; *p* = 0.027), renal insufficiency (HR, 2.36; 95% CI, 1.60–3.50; *p* < 0.001), history of previous stroke (HR, 1.60; 95% CI, 1.13–2.29; *p* = 0.009), and HFrEF (HR, 2.54; 95% CI, 1.78–3.64; *p* < 0.001) were predictors of increased risk of MACCE.

**Conclusions:**

Heart failure, rather than acute myocardial infarction, is currently the primary cause of LVT. A trend towards better prognosis in the no anticoagulation group was noted. Multivariable, matching and weighting analysis showed no improvement in prognosis with anticoagulant therapy. Our study does not negate the efficacy of anticoagulation but suggests the need to strengthen the management of anticoagulation in order to achieve better efficacy.

## 1. Introduction

Left ventricular thrombus (LVT), a severe consequence of ventricular dysfunction, is linked to a significant risk of major adverse cardiac and cerebrovascular events (MACCE) [1]. The temporal incidence of LVT after myocardial infarction may be decreasing due to improved percutaneous coronary intervention (PCI) [1, 2], however the risk of LVT cannot be overlooked [3]. Recommendations for drug treatment of LVT are based on evidence from the ST-segment elevation myocardial infarction (STEMI) guidelines in acute myocardial infarction (AMI) patients [4, 5]. However, the etiology of LVT has changed—heart failure is now the most common cause of LVT [6]. A recent scientific statement [7] recommended anticoagulation in patients with LVT in the presence of dilated cardiomyopathy for at least 3–6 months, with discontinuation if the ejection fraction improves to >35% or if major bleeding occurs. For patients with ischemic heart disease, the long-term prognosis of patients with heart failure is significantly worse than that of patients with an AMI [8]. Although Virchow’s triad is present in patients with heart failure [9], the negative results of the Zannad *et al*. [10] do not provide any justification for left ventricular ejection fraction (LVEF) ≤40% in patients who underwent anticoagulation of rivaroxaban. Compared to no anticoagulation or subtherapeutic anticoagulation, there is limited evidence that anticoagulant therapy is more likely to resolve LVT and reduce embolic risk [7]. There are still questions regarding the treatment of LVT [11]. The purpose of this study was to review the causes of LVT and changes in antithrombotic therapy over the past 12 years and assess the impact of anticoagulant treatment from a single center’s experience.

## 2. Methods

### 2.1 Study Design and Patient Population

All 1675 consecutive patients who were hospitalized with LVT at the Fuwai Hospital between January 2009 and June 2021 were included. Preliminary screening was performed using discharge diagnosis.

### 2.2 Baseline and Follow-Up

Medical reports were used to gather all patient baseline data, including long-term anticoagulant treatment before an LVT diagnosis. The type of anticoagulant used and its relationship to antiplatelet therapy received were also documented. The pattern of follow-up was consistent with Robinson *et al*. [12]. The interquartile range (IQR) for the follow-up period was 1.9–6.6 years (median, 3.8 years). The overall follow-up rate was 91.1% (1526/1675).

### 2.3 Thrombus Evaluation

Patients were diagnosed with LVT using transthoracic echocardiography, contrast-enhanced computed tomography, or cardiac magnetic resonance (CMR) imaging. A ventricular cavity with an abnormal echo mass or intensity, whose margin was distinct from the ventricular endocardium, was recognized as a ventricular thrombus. Morphological information of the thrombus including location, range of motion, mural or protruding, number and density were also recorded.

### 2.4 Endpoint Definitions

The endpoints were all-cause death, cardiovascular death, ischemic stroke, MACCE, systemic embolism (SE), and major bleeding. MACCE was a composite of end points, including cardiovascular death, ischemic stroke, and AMI. SE included ischemic stroke, AMI, or acute peripheral artery emboli (limb, renal, or digestive arteries). The occurrence of a Bleeding Academic Research Consortium (BARC) type 2, 3, or 5 bleeding was defined as major bleeding [13].

### 2.5 Statistical Analysis

All continuous variables were expressed by median and IQR, whereas categorical variables are presented as numbers and percentages. Categorical variables were compared using the χ^2^ test or Fisher’s exact test, and continuous variables were compared using the Mann-Whitney test. All *p*-values < 0.05 were considered statistically significant. Data were taken from the intention-to-treat population during the follow-up period after discharge. Statistical analyses were conducted using R 4.2.1 (R Foundation for Statistical Computing, Vienna, Austria).

Competing-risk analysis was performed in line with previous studies [14, 15]. Heterogeneity of the treatment effect for MACCE was evaluated by assessing treatment interactions across subgroups, including age, sex, BMI, LVEF, diabetes, and eGFR.

Propensity score matching (PSM) and inverse probability of treatment weighting (IPTW) were performed as sensitivity analysis. Information about matching and weighting is described in detail in the **Supplementary Methods**.

## 3. Results

### 3.1 Patient Characteristics

**Fig. 1.**
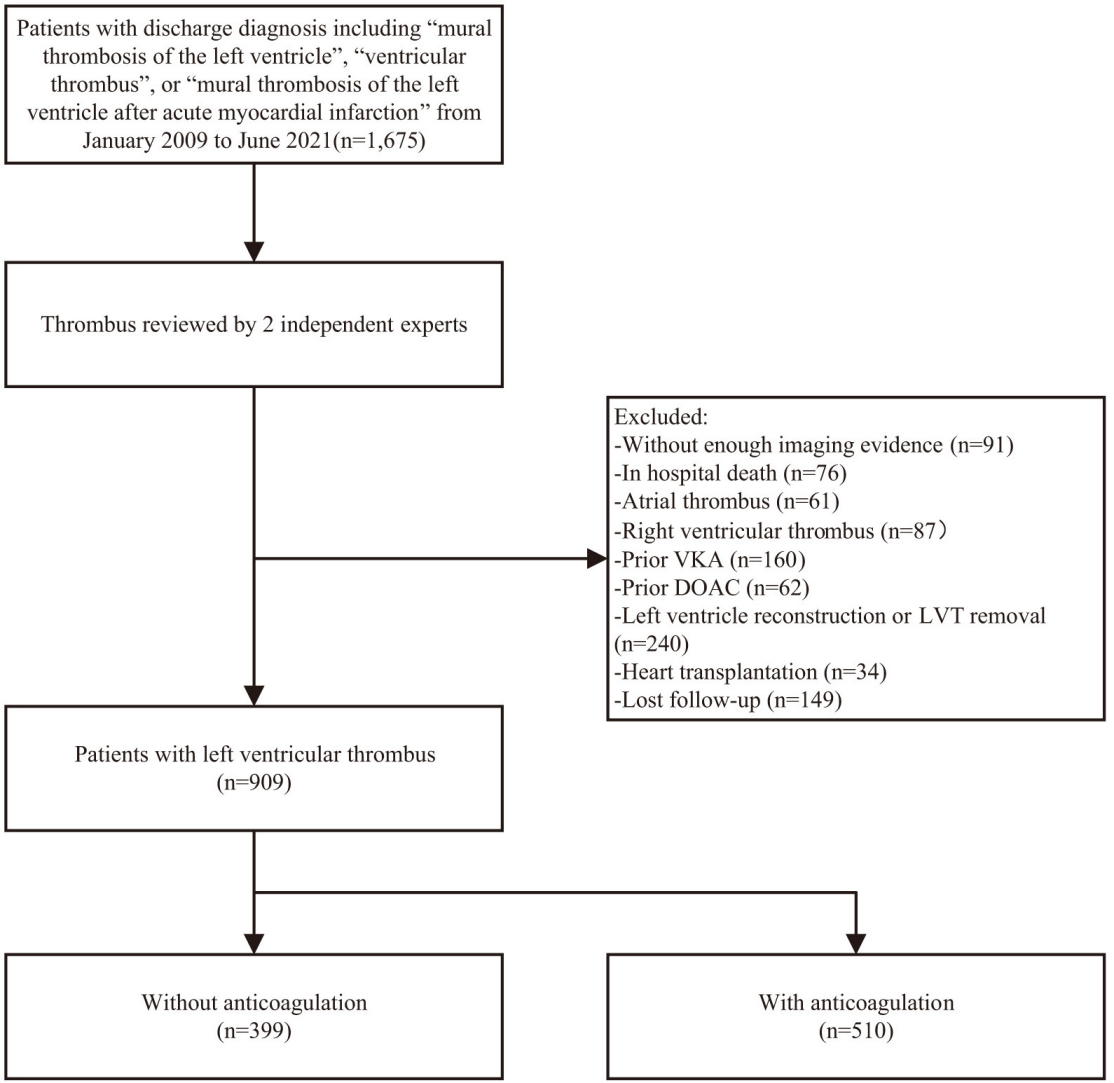
**Study flow chart.** VKA, vitamin K antagonists; DOAC, direct oral anticoagulants; LVT, left ventricular thrombus.

Among 1675 patients extracted from an electronic database according to International Classification of Diseases (ICD) codes, 909 were included in final analysis (Fig. 1). The baseline characteristics of the patients are listed in Table 1. The cohort had a median age of 55.0 (45.0–64.0) years and a high prevalence of coronary artery disease (77.2%, n = 702). One in four patients (23.9%, n = 217) had a STEMI. More than 50% of the population (60.8%, n = 553) had a reduced LVEF (LVEF ≤40%). LVT was primarily seen in the apical segments (91.5%, n = 832). A mobile LVT was present in 8.5% (n = 77) of patients, and a round LVT was observed in 61.5% (n = 559) of patients. Additionally, 51.9% (n = 472) of the patients had left ventricular aneurysms.

**Table 1.** **Baseline feature**.

		Overall	Without anticoagulation	With anticoagulation	*p* value
n		909	399	510	
Demographic					
	Age	55.0 [45.0, 64.0]	57.0 [48.0, 67.0]	53.0 [42.0, 62.0]	<0.001
	Male	753 (82.8)	337 (84.5)	416 (81.6)	0.290
	Body mass ${\mathrm{index/kg/m}}^{2}$	25.00 [22.72, 27.47]	24.97 [22.85, 27.30]	25.01 [22.61, 27.76]	0.519
Past medical history					
	Hypertension	445 (49.0)	205 (51.4)	240 (47.1)	0.220
	Diabetes mellitus	366 (40.3)	159 (39.8)	207 (40.6)	0.875
	eGFR <60 mL/min/1.73 $m^{2}$	133 (14.6)	54 (13.5)	79 (15.5)	0.463
	Peripheral artery disease	68 (7.5)	26 (6.5)	42 (8.2)	0.395
	Prior stroke	149 (16.4)	63 (15.8)	86 (16.9)	0.731
	Prior MI	472 (51.9)	248 (62.2)	224 (43.9)	<0.001
	Prior CABG	19 (2.1)	11 (2.8)	8 (1.6)	0.313
	Prior PCI	138 (15.2)	66 (16.5)	72 (14.1)	0.359
	Prior cerebral hemorrhage	6 (0.7)	4 (1.0)	2 (0.4)	0.475
	Atrial fibrillation	71 (7.8)	25 (6.3)	46 (9.0)	0.158
Underlying disease					
	Coronary artery disease	702 (77.2)	368 (92.2)	334 (65.5)	<0.001
	STEMI	217 (23.9)	125 (31.3)	92 (18.0)	<0.001
	NSTEMI	39 (4.3)	20 (5.0)	19 (3.7)	0.432
	Dilated cardiomyopathy	148 (16.3)	22 (5.5)	126 (24.7)	<0.001
	Hypertrophic cardiomyopathy	19 (2.1)	3 (0.8)	16 (3.1)	0.024
	ARVD with associated LV impairment	4 (0.4)	1 (0.3)	3 (0.6)	0.796
	Perinatal cardiomyopathy	12 (1.3)	1 (0.3)	11 (2.2)	0.027
	Restrictive cardiomyopathy	4 (0.4)	0 (0.0)	4 (0.8)	0.205
	Alcoholic cardiomyopathy	11 (1.2)	0 (0.0)	11 (2.2)	0.008
	Myocarditis	6 (0.7)	2 (0.5)	4 (0.8)	0.912
	NVM	20 (2.2)	5 (1.3)	15 (2.9)	0.135
Medications					
	Aspirin	547 (60.2)	359 (90.0)	188 (36.9)	<0.001
	Clopidogrel	452 (49.7)	269 (67.4)	183 (35.9)	<0.001
	Ticagrelor	38 (4.2)	34 (8.5)	4 (0.8)	<0.001
	DAPT	405 (44.6)	293 (73.4)	112 (22.0)	<0.001
	VKA	295 (32.5)	0 (0.0)	295 (57.8)	<0.001
	Rivaroxaban	198 (21.8)	0 (0.0)	198 (38.8)	<0.001
	Dabigatran	17 (1.9)	0 (0.0)	17 (3.3)	0.001
	DOAC	215 (23.7)	0 (0.0)	215 (42.2)	<0.001
	Antiplatelet therapy only	369 (40.6)	369 (92.5)	0 (0.0)	<0.001
	Anticoagulation only	247 (27.2)	0 (0.0)	247 (48.4)	<0.001
	Anticoagulation status				<0.001
	Dabigatran 110 mg BID	16 (1.8)	0 (0.0)	16 (3.1)	
	Rivaroxaban 2.5 mg QD	9 (1.0)	0 (0.0)	9 (1.8)	
	Rivaroxaban 5 mg QD	6 (0.7)	0 (0.0)	6 (1.2)	
	Rivaroxaban 10 mg QD	18 (2.0)	0 (0.0)	18 (3.5)	
	Rivaroxaban 15 mg QD	57 (6.3)	0 (0.0)	57 (11.2)	
	Rivaroxaban 15 mg BID	25 (2.8)	0 (0.0)	25 (4.9)	
	Rivaroxaban 20 mg QD	79 (8.7)	0 (0.0)	79 (15.5)	
	Aspirin with anticoagulant	76 (8.4)	0 (0.0)	76 (14.9)	<0.001
	Clopidogrel with anticoagulant	74 (8.1)	0 (0.0)	74 (14.5)	<0.001
	Anticoagulant with DAPT	112 (12.3)	0 (0.0)	112 (22.0)	<0.001
Imaging morphology of LVT					
	LVEDD	58.0 [53.0, 65.0]	56.0 [51.0, 60.4]	60.0 [55.0, 69.0]	<0.001
	LVEF	38.0 [29.0, 46.0]	41.0 [34.0, 48.0]	34.0 [26.0, 43.0]	<0.001
	LVEF ≤40%	553 (60.8)	198 (49.6)	355 (69.6)	<0.001
	Global hypokinesis	229 (25.2)	43 (10.8)	186 (36.5)	<0.001
	Hypokinesis	395 (43.5)	212 (53.1)	183 (35.9)	<0.001
	Akinesis	562 (61.8)	289 (72.4)	273 (53.5)	<0.001
	Apical LVT	832 (91.5)	364 (91.2)	468 (91.8)	0.866
	Round LVT	559 (61.5)	219 (54.9)	340 (66.7)	<0.001
	Mobile LVT	77 (8.5)	15 (3.8)	62 (12.2)	<0.001
	Multiple LVT	107 (11.8)	23 (5.8)	84 (16.5)	<0.001
	Calcified LVT	167 (18.4)	53 (13.3)	114 (22.4)	0.001
	LVT largest diameter/mm	23.0 [16.0, 31.0]	23.0 [17.0, 32.0]	22.0 [16.0, 31.0]	0.182
	LVT ${\mathrm{area/mm}}^{2}$	2.9 [1.6, 4.6]	2.9 [1.7, 4.5]	2.8 [1.6, 4.6]	0.906
	Left ventricular aneurysm	472 (51.9)	252 (63.2)	220 (43.1)	<0.001

Data are n/N (%) or median (IQR). ARVD, arrhythmogenic right ventricular dysplasia; CABG, coronary artery bypass grafting; DAPT, dual antiplatelet therapy; DOAC, direct oral anticoagulants; DAPT, dual antiplatelet therapy; eGFR, estimated glomerular filtration rate; LVT, left ventricular thrombus; LVEDD, left ventricular end diastolic dimension; LVEF, left ventricular ejection fraction; LV, left ventricle; MI, myocardial infarction; NSTEMI, non-ST-segment elevation myocardial infarction; NVM, noncompaction of the ventricular myocardium; PCI, percutaneous coronary intervention; STEMI, ST-segment elevation myocardial infarction; VKA, vitamin K antagonists; BID: bis in die (in Latin, twice a day); QD, quaque die (which means, in Latin, once a day).

Compared with patients without anticoagulation (n = 399), those who received anticoagulation (n = 510) at discharge were younger (53.0 vs. 57.0, *p* < 0.001), had a lower incidence of prior myocardial infarction (43.9% vs. 62.2%, *p* < 0.001), coronary artery disease (65.5% vs. 92.2%, *p* < 0.001), and STEMI (18.0% vs. 31.3%, *p* < 0.001). Additionally, the anticoagulation group had a significantly higher proportion of dilated cardiomyopathy than the non-anticoagulation group (24.7% vs. 5.5%, *p* < 0.001), with a higher proportion of global hypokinesis (36.5% vs. 10.8%, *p* < 0.001), lower LVEF (34.0 vs. 41.0, *p* < 0.001), and higher left ventricular end diastolic diameter (LVEDD) (60.0 vs. 56.0, *p* < 0.001). The anticoagulant group had a lower rate of aspirin (36.9% vs. 90.0%, *p* < 0.001) and clopidogrel (35.9% vs. 67.4%, *p* < 0.001) usage than those without anticoagulation. The anticoagulant group had a higher proportion of mobile (12.2% vs. 3.8%, *p* < 0.001), round (66.7% vs. 54.9%, *p* < 0.001), multiple (16.5% vs. 5.8%, *p* < 0.001), and calcified (22.4% vs. 13.3%, *p* = 0.001) thrombi than those without anticoagulation.

In the anticoagulation group, more than half the patients received vitamin K antagonists (VKA) (57.8%, n = 295) and the rest received direct oral anticoagulants (DOAC) (42.2%, n = 215). Rivaroxaban was the most commonly used DOAC (38.8%, n = 198), while dabigatran was used less often (3.3%, n = 17). The doses of rivaroxaban were varied: 79 patients were prescribed 20 mg once daily, 57 were prescribed 15 mg once daily, 25 were prescribed 15 mg twice daily, and 18 were prescribed 10 mg once daily.

### 3.2 Time Trends of Antithrombotic Therapy, Etiologies, and Outcomes

**Fig. 2.**
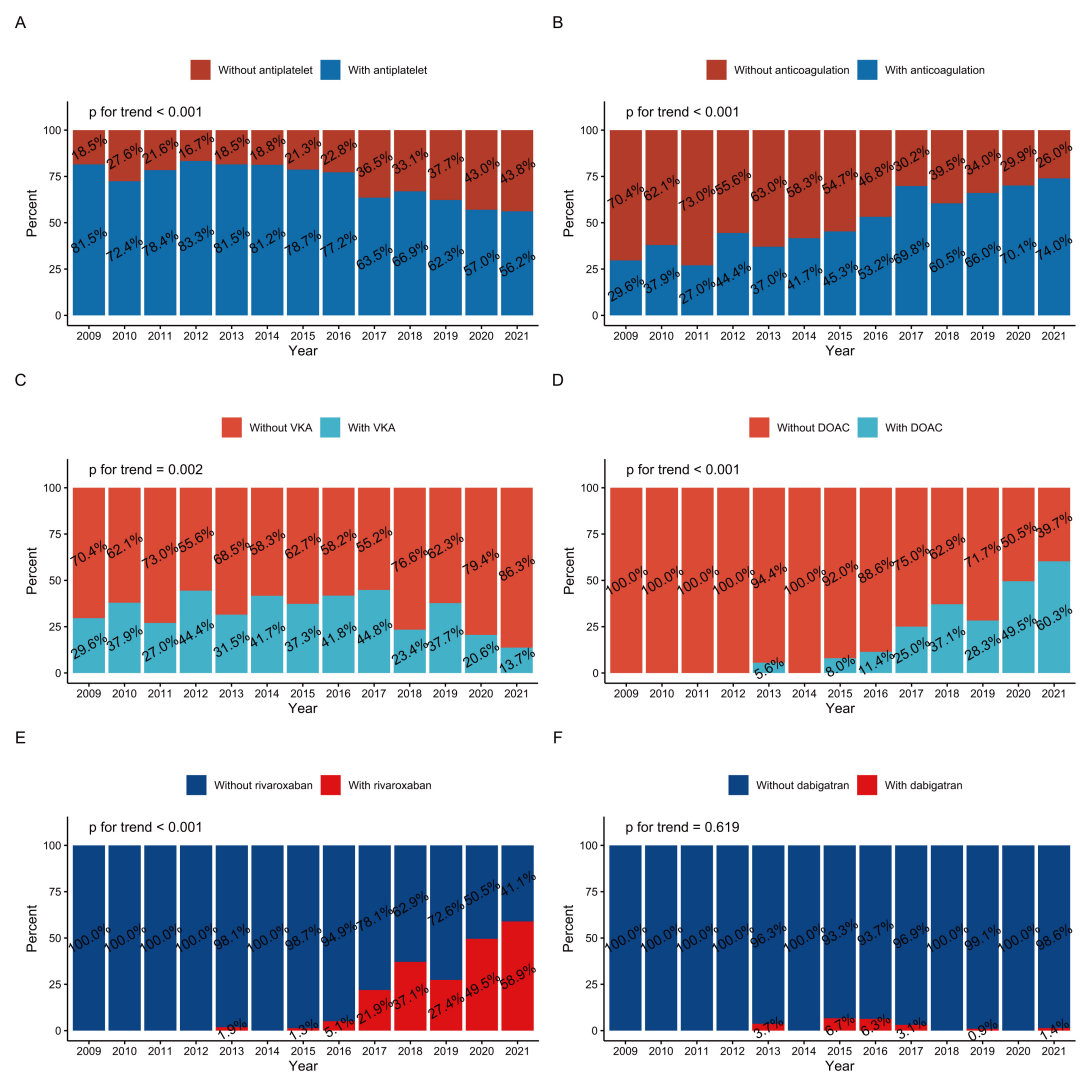
**Time trends of therapy among patients with LVT at discharge (2009–2021): (A) antiplatelet therapy, (B) oral anticoagulation therapy, (C) VKA, (D) DOAC, (E) rivaroxaban, and (F) dabigatran.** DOAC, direct oral anticoagulants; LVT, left ventricular thrombus; VKA, vitamin K antagonists.

While overall antiplatelet therapy has dramatically decreased (Fig. 2A), more patients with LVT started oral anticoagulation (OAC) in 2021 than in 2009 (74.0% vs. 29.6%, Fig. 2B). The use of VKA has dropped yearly, from a peak of 44.8% in 2017 to 13.7% in 2021 (Fig. 2C). In contrast, DOAC usage has increased, especially in the past 6 years, from 11.4% in 2016 to 60.3% in 2021 (Fig. 2D). The proportion of rivaroxaban has also rapidly increased (Fig. 2E), whereas the use of dabigatran has been low for over a decade (Fig. 2F). All p values for the trend except for dabigatran were <0.05.

Over 50% of patients with LVT had a LVEF ≤40% (**Supplementary Fig. 1A**) in each year, whereas the proportion of patients with AMI fell from 37.0% in 2009 to 19.2% in 2021 (**Supplementary Fig. 1B**). The proportion of STEMI also decreased significantly (**Supplementary Fig. 1C**, *p* value for trend <0.001), while the proportion of Non-ST-segment elevated myocardial infarction (NSTEMI) increased (**Supplementary Fig. 1D**, *p* value for trend = 0.013).

As shown in **Supplementary Fig. 2**, patients discharged between 2016 and 2021 had a higher risk of stroke (*p* = 0.012), SE (*p* = 0.001), and major bleeding (*p* = 0.025) than those discharged between 2009 and 2015. There were no significant differences in all-cause mortality (*p* = 0.414), cardiovascular death (*p *= 0.561), or MACCE (*p* = 0.101) between the two groups.

### 3.3 Competing Risks of Terminal Outcomes

**Fig. 3.**
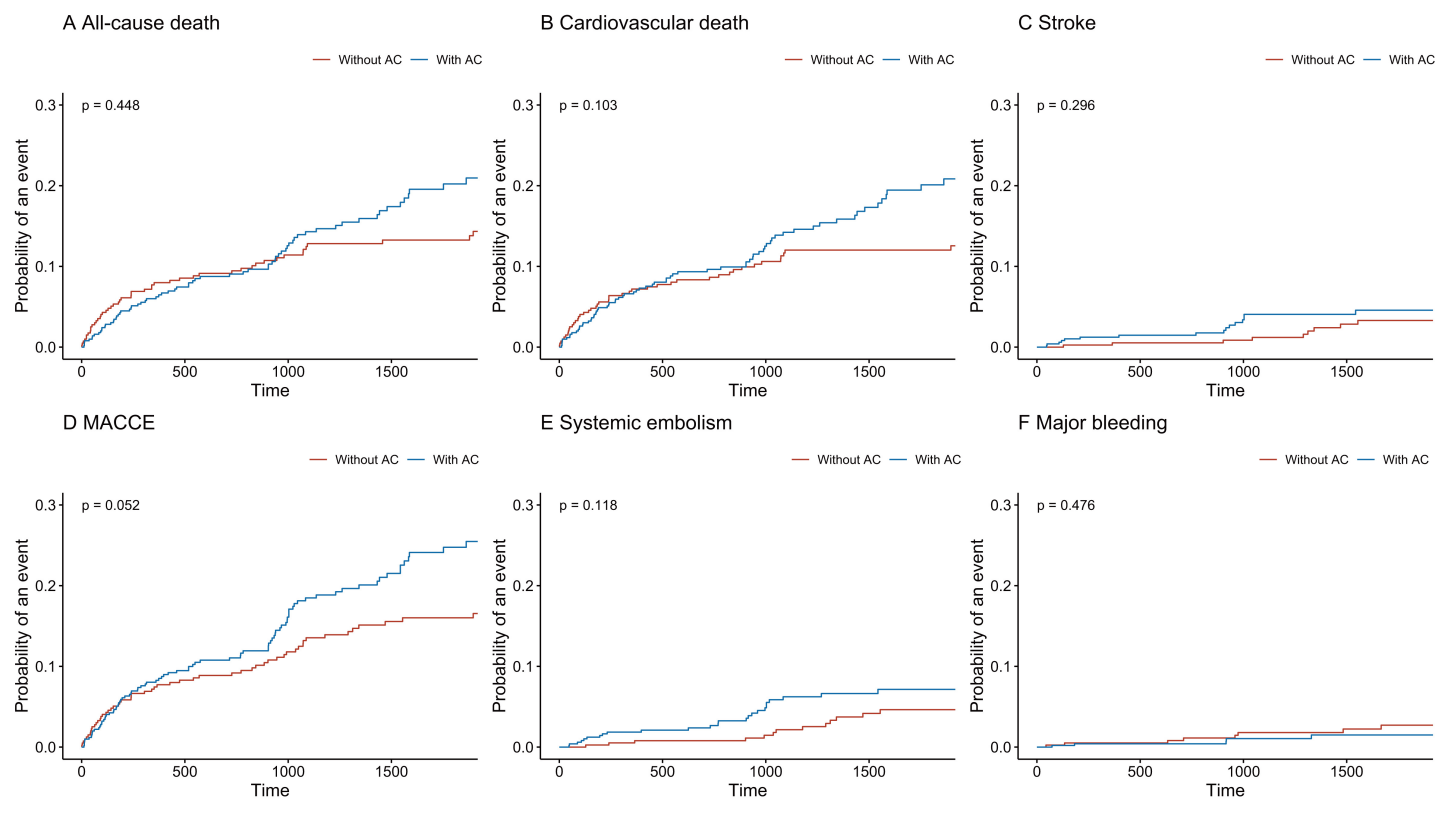
**Time-to-event curves according to anticoagulation treatment.** AC, anticoagulation; MACCE, major adverse cardiac and cerebrovascular events.

As shown in Table 2, all-cause mortality was 17.3% (n = 157). The incidence of cardiovascular death and stroke was 16.0% (n = 145) and 3.3% (n = 30), respectively. Moreover, MACCE and SE occurred in 19.8% (n = 180) and 5.1% (n = 46) of patients, respectively, while major bleeding (BARC ≥2) events occurred in 1.7% (n = 15) of patients. The incidence of MACCE (Fig. 3D) was higher in the anticoagulation group than in the group with no anticoagulation, but there was no statistical difference (*p* = 0.052). In the multivariable competing risk regression analysis (Table 2), there was no significant relationship between anticoagulation and all six endpoints (all *p* > 0.05).

**Table 2.** **Outcomes in the whole population and according to the anticoagulation therapy at discharge**.

	Overall (n = 909)	Without anticoagulation (n = 399)	With anticoagulation (n = 510)	Multivariable analysis		PSM analysis		IPTW analysis	
				Hazard ratio (95% CI)	*p* values	Hazard ratio (95% CI)	*p* values	Hazard ratio (95% CI)	*p* values
All-cause death	157 (17.3%)	77 (19.3)	80 (15.7)	0.96 (0.67–1.38)	0.800	0.71 (0.45–1.12)	0.140	0.83 (0.59–1.18)	0.307
Cardiovascular death	145 (16.0%)	65 (16.3)	80 (15.7)	1.05 (0.72–1.53)	0.800	0.74 (0.47–1.18)	0.200	0.91 (0.63–1.31)	0.616
Stroke	30 (3.3%)	13 (3.3)	17 (3.3)	1.62 (0.76–3.45)	0.200	1.72 (0.66–4.47)	0.300	1.59 (0.74–3.41)	0.232
MACCE	180 (19.8%)	81 (20.3)	99 (19.4)	1.15 (0.82–1.61)	0.400	0.88 (0.59–1.32)	0.500	1.03 (0.74–1.44)	0.846
Systemic embolism	46 (5.1%)	19 (4.8)	27 (5.3)	1.69 (0.92–3.13)	0.093	1.40 (0.66–2.97)	0.400	1.81 (0.97–3.39)	0.062
Major bleeding	15 (1.7%)	9 (2.3)	6 (1.2)	0.77 (0.29–2.03)	0.600	0.49 (0.10–2.48)	0.400	0.78 (0.26–2.34)	0.663

Values are n (%). MACCE, major adverse cardiac and cerebrovascular events; CI, confidence interval; PSM, propensity score matching; IPTW, inverse probability of treatment weighting.

After 1:1 PSM, 514 patients (anticoagulation, 257; no anticoagulation, 257) were selected (**Supplementary Table 1**). Ultimately, 905 patients (anticoagulation, 510.38; no anticoagulation, 394.62) were chosen after IPTW (**Supplementary Table 2**). All baseline variables included in the matching and weighting process became balanced (standardized mean difference (SMD) <0.2, **Supplementary Fig. 3**). After PSM and IPTW, competing risk regression analysis (Table 2) indicated that anticoagulation did not benefit patients in all six endpoints, including all-cause death.

**Fig. 4.**
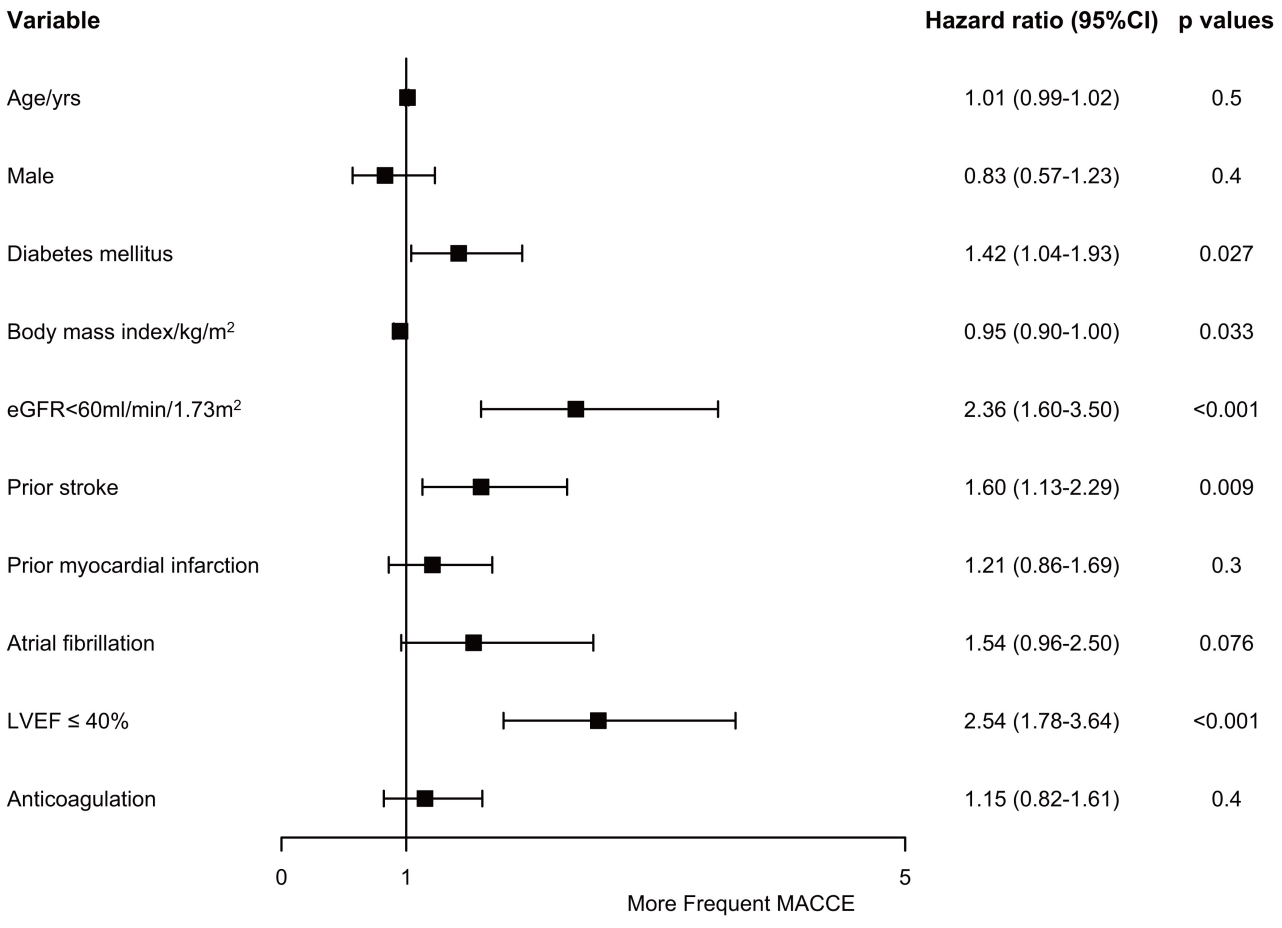
**Risk factors of major adverse cardiac and cerebrovascular events.** CI, confidence interval; eGFR, estimated glomerular filtration rate; LVEF, left ventricular ejection fraction; MACCE, major adverse cardiac and cerebrovascular events.

Multivariable analysis concludes diabetes mellitus (hazard ratio (HR), 1.42; 95% confidence interval (CI), 1.04–1.93; *p* = 0.027), estimated glomerular filtration rate (eGFR) <60 mL/min/1.73 $m^{2}$ (HR, 2.36; 95% CI, 1.60–3.50; *p* < 0.001), history of previous stroke (HR, 1.60; 95% CI, 1.13–2.29; *p* = 0.009), and LVEF ≤40% (HR, 2.54; 95% CI, 1.78–3.64; *p* < 0.001) were independently linked to a higher risk of MACCE (Fig. 4). It should be noted that anticoagulant therapy (HR, 1.15; 95% CI, 0.82–1.61; *p* = 0.4) was not independently associated with MACCE in our study.

### 3.4 Sensitivity Analysis

**Fig. 5.**
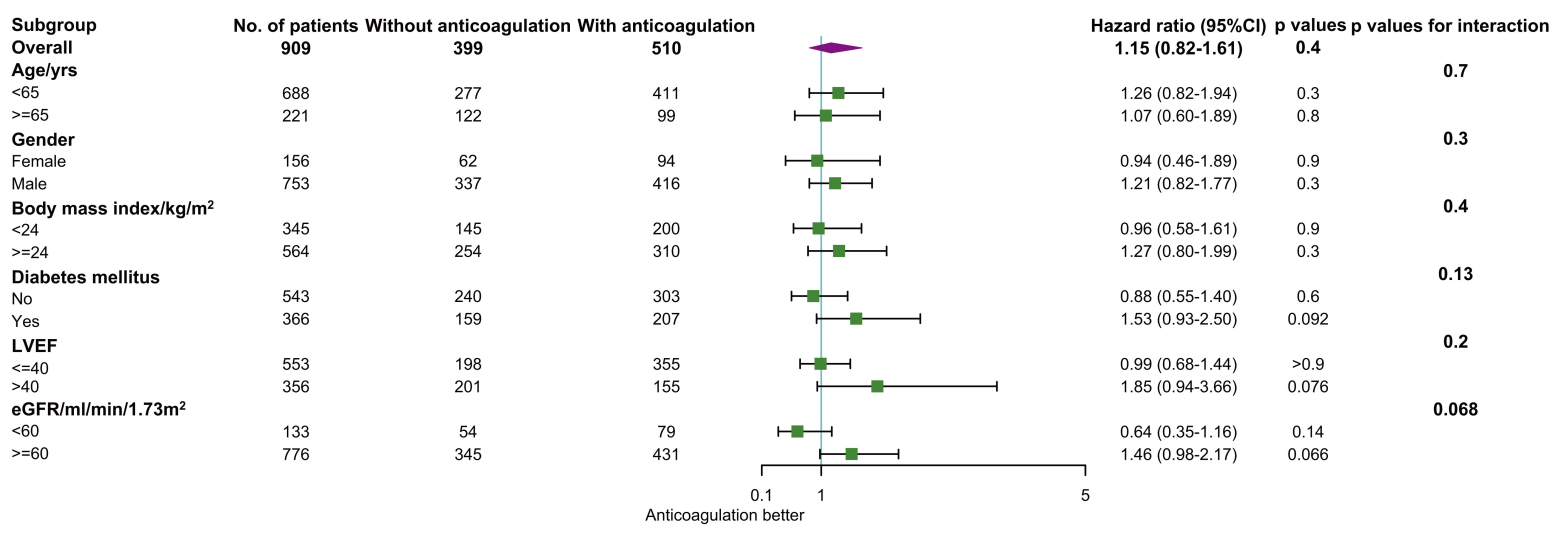
**Relationship between anticoagulation therapy and risk of major adverse cardiac and cerebrovascular events in subgroup analyses.** CI, confidence interval; LVEF, left ventricular ejection fraction; eGFR, estimated glomerular filtration rate.

The results were in line with the overall efficacy finding across all subgroups (Fig. 5). None of the interactions were significant. Surprisingly, in patients with LVEF >40%, anticoagulant therapy (HR, 1.85; 95% CI, 0.94–3.66; *p* = 0.076, *p* for interaction = 0.2) was not independently associated with MACCE.

**Fig. 6.**
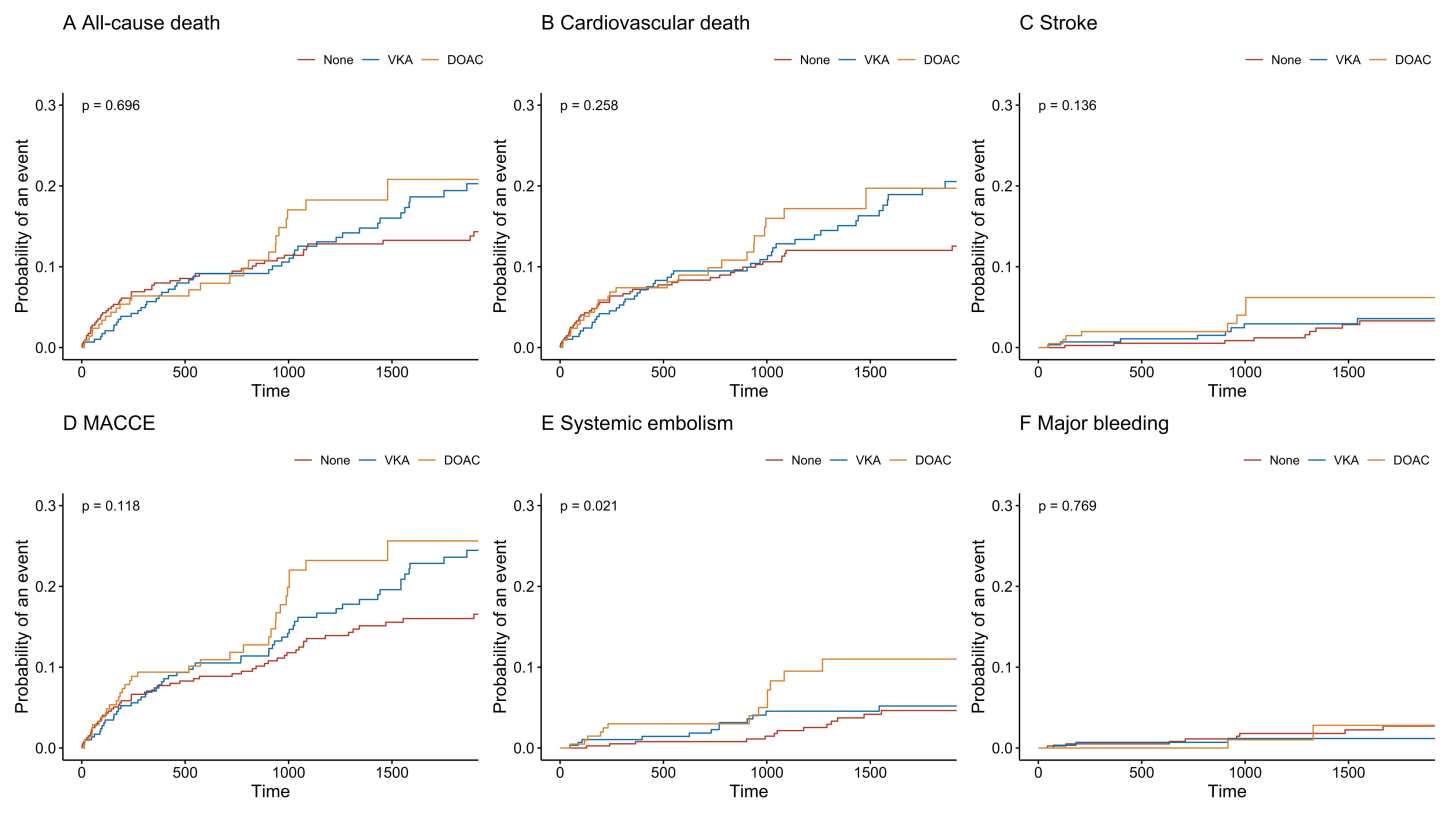
**Time-to-event curves according to anticoagulation treatment (None vs. VKA vs. DOAC).** VKA, Vitamin K antagonists; DOAC, direct oral anticoagulants; MACCE, major adverse cardiac and cerebrovascular events.

As shown in Fig. 6, the direct oral anticoagulants (DOAC) group had a higher risk of SE than the other two groups (*p* = 0.021).

## 4. Discussion

To the best of our knowledge, our study is the largest LVT cohort. The study’s key conclusions include the following: (i) heart failure with reduced ejection fraction overtook AMI as the leading cause of LVT; (ii) currently, an increasing proportion of patients with LVT are receiving anticoagulant therapy at discharge; (iii) since 2020, rivaroxaban has displaced warfarin as the most popular OAC for LVT patients; (iv) a trend towards better prognosis in the no anticoagulation group can be noted from survival curves; (v) from multivariable, matching and weighting analysis, no improvement in prognosis was observed with anticoagulant therapy; (vi) diabetes mellitus, eGFR <60 mL/min/1.73 $m^{2}$, prior stroke, and LVEF ≤40% were predictors of increased risk of MACCE.

Previous research has often added the qualifier of AMI to LVT [1, 2, 16, 17] while ignoring ≥50% of the heart failure population with LVT. Our study confirms that heart failure, rather than AMI, is the most common cause of LVT. McCarthy *et al*. [6] also reported this finding. There is more robust evidence for anticoagulation in LVT caused by AMI. However, in our study, the actual incidence of anticoagulation was lower in patients with AMI than in those without AMI (43.3% vs. 61.1%, *p* < 0.001). A potential explanation is the increased risk of bleeding caused by combined antiplatelet and anticoagulant treatment. Nearly half (399/909) of patients with a newly diagnosed LVT were not treated with OAC on discharge in our study. In European [16] and American [18] studies, almost all patients with LVT were treated with anticoagulation. This reflects the poor anticoagulation status of LVT in China. Anticoagulant therapy for LVT has not received much attention. The good news is that this proportion is increasing year by year. Prior research has shown that DOAC comprise an increasing fraction of OAC used to treat LVT, whereas VKA remain the most commonly used agent [1, 6, 12, 16]. The same trend was observed in our data, but our off-label application of DOAC was more aggressive, and rivaroxaban exceeded warfarin.

Lemaitre *et al*. [19] found all-cause mortality occurred in 10% of patients with LVT and heart failure, while symptomatic emboli occurred in 15% and major bleeding occurred in 5% during a median follow-up of 8.7 years. This differs from the findings in our current study. In our cohort, the risk of SE and major bleeding was lower, while the risk of all-cause mortality was higher. Compared with the study findings of Lattuca *et al*. [18], LVEF was similar in our population (31.9% vs. 38.0%), but the proportion of mobile LVT was lower (34.6% vs. 8.5%), and the proportion of calcified LVT (1.3% vs. 18.4%) was higher. The risk of embolism in patients with LVT is closely related to two morphologic features: movement and protrusion [2]. Differences in LVT characteristics may be a possible explanation for the lower risk of embolism in our population. The major bleeding was higher in the no anticoagulation arm (2.3% vs. 1.2%). This may be due to the fact that the proportion of dual antiplatelet therapy in the no-anticoagulation group was much higher than that in the anticoagulation group (73.4% vs. 22.0%, *p* < 0.001). Our results corroborate the findings of Lee *et al*. [20], who found that in patients with LV aneurysms and LVT, the benefit of oral anticoagulation with warfarin was not observed (HR, 1.38; 95% CI, 0.32–5.97; *p* = 0.66). A short course of low-dose rivaroxaban helps prevent LVT formation in patients with an anterior STEMI after primary PCI [21]. This finding may be explained by the fact that all patients received PCI and had a relatively normal LVEF (54%). A retrospective study from Croatia [22] involving more than 4000 patients with COVID-19 found that arterial thrombi were independently associated with less severe COVID-19 but higher comorbidity burden. Since our study covered a longer time period, we did not consider the influence of COVID-19 patients on thrombotic events.

Our cohort is the largest LVT cohort reported, allowing us to perform some sensitivity analyses. With a prevalence inversely associated with LVEF [23], LVT has been documented in 11–44% of patients with heart failure [24]. Anticoagulation failed to produce positive results, even in patients with LVEF >40%. Anticoagulation did not seem to improve cardiovascular outcomes for patients with LVT. There are several possible explanations. First, the baseline features of the anticoagulant and non-anticoagulant groups were different. The anticoagulation group had a significantly higher proportion of dilated cardiomyopathy than the non-anticoagulation group with a lower LVEF. In terms of thrombosis morphology, the anticoagulant group had a higher proportion of mobile, round and multiple thrombi than those without anticoagulation. In short, patients in the anticoagulant group had worse cardiac function and a higher risk of embolic thrombi. Therefore, it is not surprising that a clear trend towards a better prognosis in the non-anticoagulant group has been observed (Fig. 3). Multivariable, matching and weighting analysis revealed there was no significant difference in prognosis between the two groups. A second possible explanation is that different types and dosages of anticoagulants may impair their effectiveness. Our study confirms that DOAC is used in a variety of doses to treat LVT. In addition, we found the DOAC group had a higher risk of SE than the other two groups. Low doses of DOAC increase mortality and the risk of embolism without reducing the risk of bleeding [25, 26]. Third, the time in the therapeutic range (TTR) of warfarin and thrombus status are unknown, and these factors are closely related to prognosis [7]. When individuals with LVT don’t keep their international normalized ratio (INR) at a therapeutic level (TTR <50%), they seem to be at a higher risk for stroke [17]. Whole LVT regression, according to Lattuca *et al*. [18], was linked to lower mortality. We did not observe a greater benefit in the anticoagulation group. Our study does not negate the efficacy of anticoagulation but suggests the need to strengthen the management of anticoagulation in order to achieve better efficacy.

## 5. Limitations

This study included the largest cohort of LVT patients to date and was a retrospective observational analysis. However, this study had several limitations. The main limitation of this study is that it is a retrospective study. We attempted to collect various factors that might influence prognosis for multivariate analysis. However, data on medication adherence and switching medications were not collected during the follow-up. In addition, imaging follow-up data on patients were difficult to obtain, so we did not conduct a relevant analysis. Our results should be viewed as exploratory because we realize that the comparison between the two groups was constrained by the disparate baseline characteristics. We cannot conclude whether anticoagulation improves cardiovascular outcomes for patients with LVT. Our study only suggests that the management of anticoagulation should be strengthened to achieve better results. Not all of the patients in our study who had LVT as determined by different imaging tests had a CMR. This may introduce heterogeneity issues. Finally, the low incidence of embolism and bleeding in our cohort may limit the validity of the findings in other races. Asian populations have a different risk of bleeding and ischemia, as demonstrated in the coronary heart disease population [27].

## 6. Conclusions

In this retrospective analysis, we examined the temporal patterns in the causes, courses of therapy, and results among Asian LVT patients. We found that heart failure, rather than AMI, was the primary cause of LVT. Additionally, there has been an increase in OAC utilization among patients discharged with LVT. Moreover, rivaroxaban has surpassed warfarin as the most popular first-line anticoagulant among patients with LVT since 2020. A trend towards better prognosis in the no anticoagulation group was noted from the survival curves. From multivariable, matching and weighting analysis, no improvement in prognosis was observed with anticoagulant therapy. Our study does not negate the efficacy of anticoagulation but suggests the need to strengthen the management of anticoagulation in order to achieve better efficacy.

## Data Availability

The data used in our study is not publicly available but is available from corresponding author on reasonable request.

## Abbreviations

AMI, acute myocardial infarction; DOAC, direct oral anticoagulants; LVT, left ventricular thrombus; LVEDD, left ventricular end diastolic dimension; LVEF, left ventricular ejection fraction; MACCE, major adverse cardiac and cerebrovascular events; PCI, percutaneous coronary intervention; STEMI, ST-segment elevation myocardial infarction; VKA, vitamin K antagonists.

## Supplementary Material

Supplementary material associated with this article can be found, in the online version, at https://doi.org/10.31083/j.rcm2410298.
