# Supplementary material for: Time Trends of Etiology, Treatment, and Long-Term Outcomes Among Patients with Left Ventricular Thrombus
Source: Rev Cardiovasc Med. 2023 Oct 20;24(10):298. doi: 10.31083/j.rcm2410298 (PMC11273134; doi:10.31083/j.rcm2410298)
Supplement: Supplementary file 1 [file 2153-8174-24-10-298-s1.zip › v0-Supplementary material.docx]

Supplementary Materials

# 1. Supplementary Methods

We performed matching using the nearest-neighbor method with a ratio of 1:1. The propensity score estimation was identified by a multivariable logistic regression. The variables included in the model estimating propensity score: age, gender, body mass index, hypertension, diabetes mellitus, eGFR<60ml/min/1.73m^2^, peripheral artery disease, prior stroke, prior MI, prior CABG, prior PCI, prior cerebral hemorrhage, atrial fibrillation, coronary artery disease, STEMI, NSTEMI, dilated cardiomyopathy, hypertrophic cardiomyopathy, ARVD with associated LV impairment, perinatal cardiomyopathy, restrictive cardiomyopathy, alcoholic cardiomyopathy, myocarditis, NVM, LVEDD, LVEF, LVEF<=40%, global hypokinesis, hypokinesis, akinesis, apical LVT, round LVT, mobile LVT, multiple LVT, calcified LVT, LVT largest diameter, LVT area, left ventricular aneurysm. The standardized mean difference (SMD) was used to evaluate the availability of PSM in reducing the baseline discrepancy between the two groups. An SMD<20% indicated successful PSM.

# 2. Supplementary Tables

Supplemental Table 1. Baseline features according to the anticoagulation therapy at discharge after propensity score matching.

|  | Without anticoagulation | With anticoagulation | P value | SMD |
| --- | --- | --- | --- | --- |
| n | 257 | 257 |  |  |
| **Demographic** |  |  |  |  |
| Age | 57.00 [46.00, 66.00] | 56.00 [46.00, 65.00] | 0.593 | 0.063 |
| Male | 212 (82.5) | 214 (83.3) | 0.907 | 0.021 |
| Body mass index/kg/m^2^ | 25.05 [23.03, 27.41] | 25.06 [22.60, 27.44] | 0.874 | 0.036 |
| **Past medical history** |  |  |  |  |
| Hypertension | 133 (51.8) | 132 (51.4) | 1 | 0.008 |
| Diabetes mellitus | 104 (40.5) | 107 (41.6) | 0.858 | 0.024 |
| eGFR<60 ml/min/1.73m^2^ | 33 (12.8) | 36 (14.0) | 0.796 | 0.034 |
| Peripheral artery disease | 17 (6.6) | 17 (6.6) | 1 | <0.001 |
| Prior stroke | 44 (17.1) | 43 (16.7) | 1 | 0.01 |
| Prior MI | 160 (62.3) | 154 (59.9) | 0.651 | 0.048 |
| Prior CABG | 5 (1.9) | 5 (1.9) | 1 | <0.001 |
| Prior PCI | 46 (17.9) | 41 (16.0) | 0.638 | 0.052 |
| Prior cerebral hemorrhage | 0 (0.0) | 0 (0.0) | NA | <0.001 |
| Atrial fibrillation | 18 (7.0) | 20 (7.8) | 0.866 | 0.03 |
| **Underlying disease** |  |  |  |  |
| Coronary artery disease | 231 (89.9) | 226 (87.9) | 0.574 | 0.062 |
| STEMI | 67 (26.1) | 71 (27.6) | 0.765 | 0.035 |
| NSTEMI | 12 (4.7) | 15 (5.8) | 0.693 | 0.052 |
| Dilated cardiomyopathy | 20 (7.8) | 18 (7.0) | 0.866 | 0.03 |
| Hypertrophic cardiomyopathy | 3 (1.2) | 3 (1.2) | 1 | <0.001 |
| ARVD with associated LV impairment | 0 (0.0) | 0 (0.0) | NA | <0.001 |
| Perinatal cardiomyopathy | 1 (0.4) | 2 (0.8) | 1 | 0.051 |
| Restrictive cardiomyopathy | 0 (0.0) | 0 (0.0) | NA | <0.001 |
| Alcoholic cardiomyopathy | 0 (0.0) | 0 (0.0) | NA | <0.001 |
| Myocarditis | 1 (0.4) | 3 (1.2) | 0.616 | 0.089 |
| NVM | 5 (1.9) | 7 (2.7) | 0.77 | 0.052 |
| **Imaging morphology of LVT** |  |  |  |  |
| LVEDD | 57.00 [52.00, 62.00] | 57.00 [52.00, 61.00] | 0.553 | 0.062 |
| LVEF | 40.00 [33.00, 47.00] | 40.00 [33.00, 48.00] | 0.77 | 0.019 |
| LVEF<=40% | 148 (57.6) | 140 (54.5) | 0.534 | 0.063 |
| Global hypokinesis | 38 (14.8) | 40 (15.6) | 0.902 | 0.022 |
| Hypokinesis | 130 (50.6) | 125 (48.6) | 0.724 | 0.039 |
| Akinesis | 184 (71.6) | 183 (71.2) | 1 | 0.009 |
| Apical LVT | 239 (93.0) | 237 (92.2) | 0.866 | 0.03 |
| Round LVT | 149 (58.0) | 155 (60.3) | 0.654 | 0.048 |
| Mobile LVT | 11 (4.3) | 12 (4.7) | 1 | 0.019 |
| Multiple LVT | 18 (7.0) | 19 (7.4) | 1 | 0.015 |
| Calcified LVT | 36 (14.0) | 38 (14.8) | 0.9 | 0.022 |
| LVT largest diameter/mm | 24.00 [17.00, 33.00] | 22.00 [16.00, 32.00] | 0.306 | 0.045 |
| LVT area/mm^2^ | 3.00 [1.68, 4.94] | 2.86 [1.60, 4.80] | 0.631 | 0.034 |
| Left ventricular aneurysm | 158 (61.5) | 161 (62.6) | 0.856 | 0.024 |

Data are n/N (%) or median (IQR). eGFR=estimated glomerular filtration rate. MI=myocardial infarction. CABG=coronary artery bypass grafting. PCI=percutaneous coronary intervention. STEMI=ST-segment elevation myocardial infarction. NSTEMI=non-ST-segment elevation myocardial infarction. ARVD=arrhythmogenic right ventricular dysplasia. NVM=noncompaction of the ventricular myocardium. LVT=left ventricular thrombus. LVEDD=left ventricular end diastolic dimension. LVEF=left ventricular ejection fraction. LVA=left ventricle aneurysms.

Supplementary Table 2. Baseline features in the whole population and according to the anticoagulation therapy at discharge after inverse probability of treatment weighting.

|  | Without anticoagulation | With anticoagulation | P value | SMD |
| --- | --- | --- | --- | --- |
| n | 394.62 | 510.38 |  |  |
| **Demographic** |  |  |  |  |
| Age | 54.00 [44.00, 64.01] | 54.05 [45.00, 64.00] | 0.943 | 0.002 |
| Male | 319.4 (80.9) | 419.0 (82.1) | 0.735 | 0.03 |
| Body mass index/kg/m^2^ | 25.14 [22.95, 27.43] | 24.91 [22.59, 27.52] | 0.664 | 0.007 |
| **Past medical history** |  |  |  |  |
| Hypertension | 179.7 (45.5) | 247.8 (48.5) | 0.448 | 0.06 |
| Diabetes mellitus | 159.1 (40.3) | 204.7 (40.1) | 0.961 | 0.004 |
| eGFR<60 ml/min/1.73m^2^ | 63.8 (16.2) | 76.5 (15.0) | 0.72 | 0.032 |
| Peripheral artery disease | 25.0 (6.3) | 37.7 (7.4) | 0.55 | 0.041 |
| Prior stroke | 69.7 (17.7) | 82.7 (16.2) | 0.634 | 0.039 |
| Prior MI | 202.6 (51.3) | 260.4 (51.0) | 0.938 | 0.006 |
| Prior CABG | 8.1 (2.1) | 9.7 (1.9) | 0.87 | 0.011 |
| Prior PCI | 58.2 (14.8) | 75.6 (14.8) | 0.98 | 0.002 |
| Prior cerebral hemorrhage | 2.8 (0.7) | 3.9 (0.8) | 0.931 | 0.007 |
| Atrial fibrillation | 29.4 (7.5) | 38.4 (7.5) | 0.976 | 0.003 |
| **Underlying disease** |  |  |  |  |
| Coronary artery disease | 297.7 (75.4) | 392.9 (77.0) | 0.717 | 0.037 |
| STEMI | 95.8 (24.3) | 123.6 (24.2) | 0.984 | 0.001 |
| NSTEMI | 16.2 (4.1) | 21.9 (4.3) | 0.903 | 0.008 |
| Dilated cardiomyopathy | 61.8 (15.7) | 83.1 (16.3) | 0.863 | 0.017 |
| Hypertrophic cardiomyopathy | 16.2 (4.1) | 10.8 (2.1) | 0.328 | 0.114 |
| ARVD with associated LV impairment | 1.3 (0.3) | 2.2 (0.4) | 0.837 | 0.015 |
| Perinatal cardiomyopathy | 3.3 (0.8) | 6.7 (1.3) | 0.664 | 0.046 |
| Restrictive cardiomyopathy | 0.0 (0.0) | 2.2 (0.4) | 0.081 | 0.094 |
| Alcoholic cardiomyopathy | 0.0 (0.0) | 6.2 (1.2) | 0.004 | 0.156 |
| Myocarditis | 3.0 (0.8) | 3.8 (0.7) | 0.978 | 0.002 |
| NVM | 16.0 (4.0) | 12.5 (2.4) | 0.356 | 0.09 |
| **Imaging morphology of LVT** |  |  |  |  |
| LVEDD | 58.00 [52.00, 65.00] | 58.00 [52.00, 65.00] | 0.952 | 0.013 |
| LVEF | 38.00 [30.00, 46.00] | 38.00 [29.00, 45.62] | 0.961 | 0.011 |
| LVEF<=40% | 242.1 (61.3) | 312.2 (61.2) | 0.965 | 0.003 |
| Global hypokinesis | 105.4 (26.7) | 129.5 (25.4) | 0.751 | 0.031 |
| Hypokinesis | 172.5 (43.7) | 225.4 (44.2) | 0.907 | 0.009 |
| Akinesis | 241.2 (61.1) | 315.1 (61.7) | 0.881 | 0.013 |
| Apical LVT | 365.8 (92.7) | 470.7 (92.2) | 0.805 | 0.018 |
| Round LVT | 244.3 (61.9) | 314.5 (61.6) | 0.938 | 0.006 |
| Mobile LVT | 30.4 (7.7) | 43.1 (8.4) | 0.789 | 0.027 |
| Multiple LVT | 45.5 (11.5) | 60.6 (11.9) | 0.916 | 0.011 |
| Calcified LVT | 65.9 (16.7) | 93.7 (18.4) | 0.611 | 0.044 |
| LVT largest diameter/mm | 23.00 [17.00, 32.00] | 22.00 [16.00, 31.00] | 0.386 | 0.012 |
| LVT area/mm^2^ | 3.08 [1.68, 4.61] | 2.82 [1.60, 4.60] | 0.511 | 0.002 |
| Left ventricular aneurysm | 205.7 (52.1) | 267.6 (52.4) | 0.942 | 0.006 |

Data are n/N (%) or median (IQR). eGFR=estimated glomerular filtration rate. MI=myocardial infarction. CABG=coronary artery bypass grafting. PCI=percutaneous coronary intervention. STEMI=ST-segment elevation myocardial infarction. NSTEMI=non-ST-segment elevation myocardial infarction. ARVD=arrhythmogenic right ventricular dysplasia. NVM=noncompaction of the ventricular myocardium. LVT=left ventricular thrombus. LVEDD=left ventricular end diastolic dimension. LVEF=left ventricular ejection fraction. LVA=left ventricle aneurysms.

# 3. Supplementary Figures


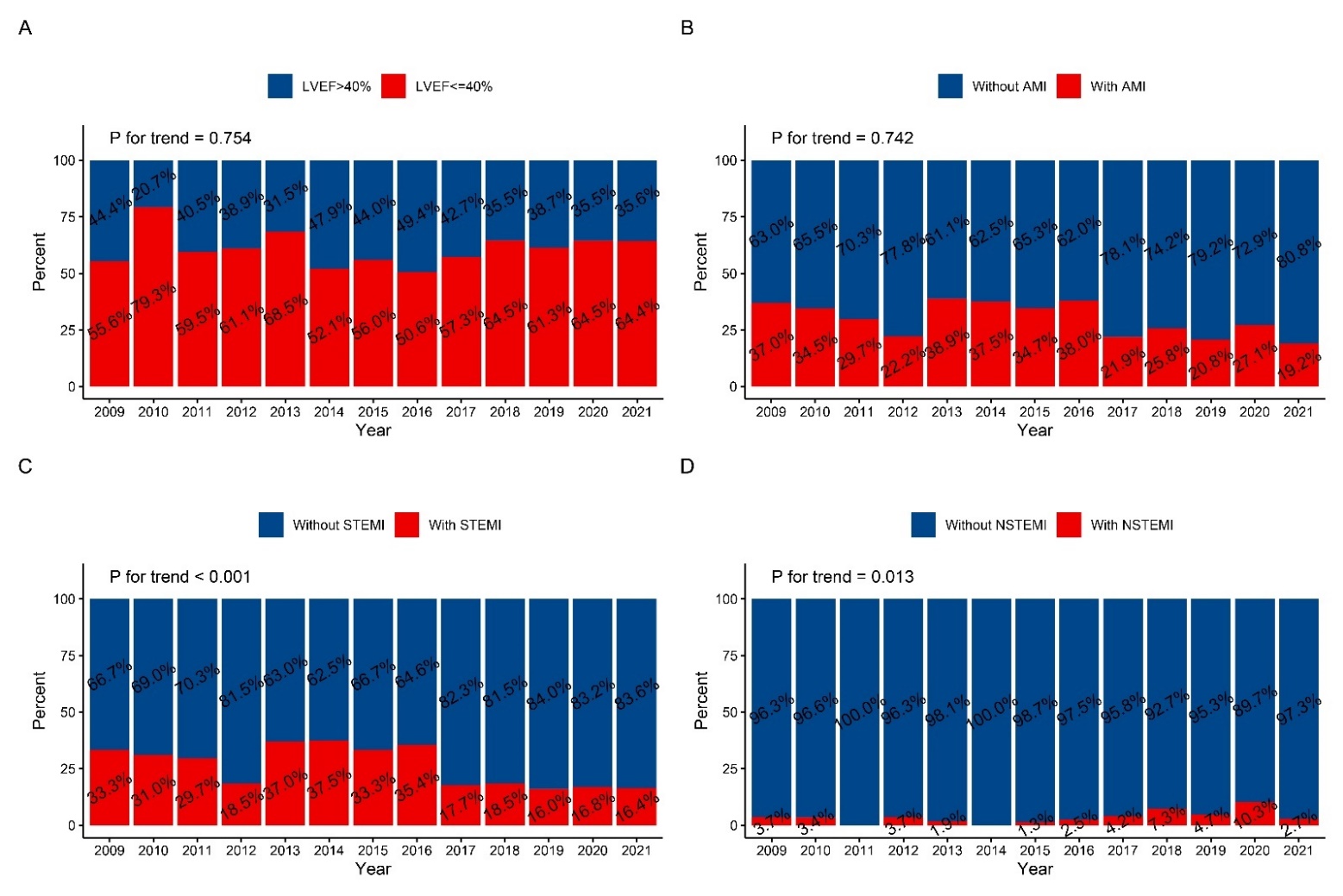


**Supplementary Fig. 1. Time trends of etiologies among patients with LVT from 2009 to 2021.** (A) Time trends of LVEF<=40%; (B) Time trends of AMI; (C) Time trends of STEMI; (D) Time trends of NSTEMI. LVT=left ventricular thrombus. LVEF=left ventricular ejection fraction. AMI=acute myocardial infarction. STEMI=ST-segment elevation myocardial infarction. NSTEMI=non–ST-segment elevation myocardial infarction.


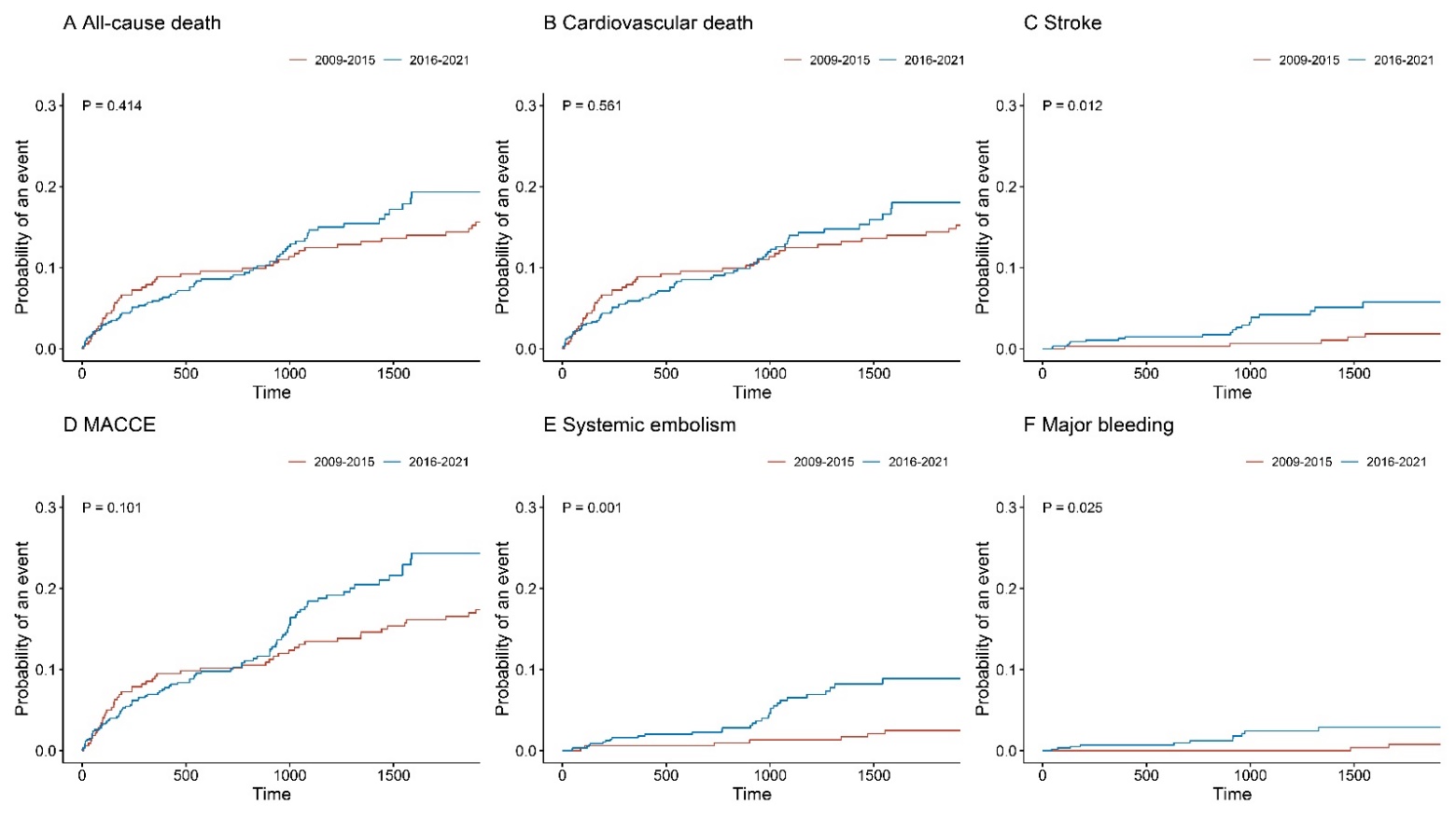


**Supplementary Fig. 2.** **Time-to-event curves according to discharge year.** MACCE=major adverse cardiac and cerebrovascular events.


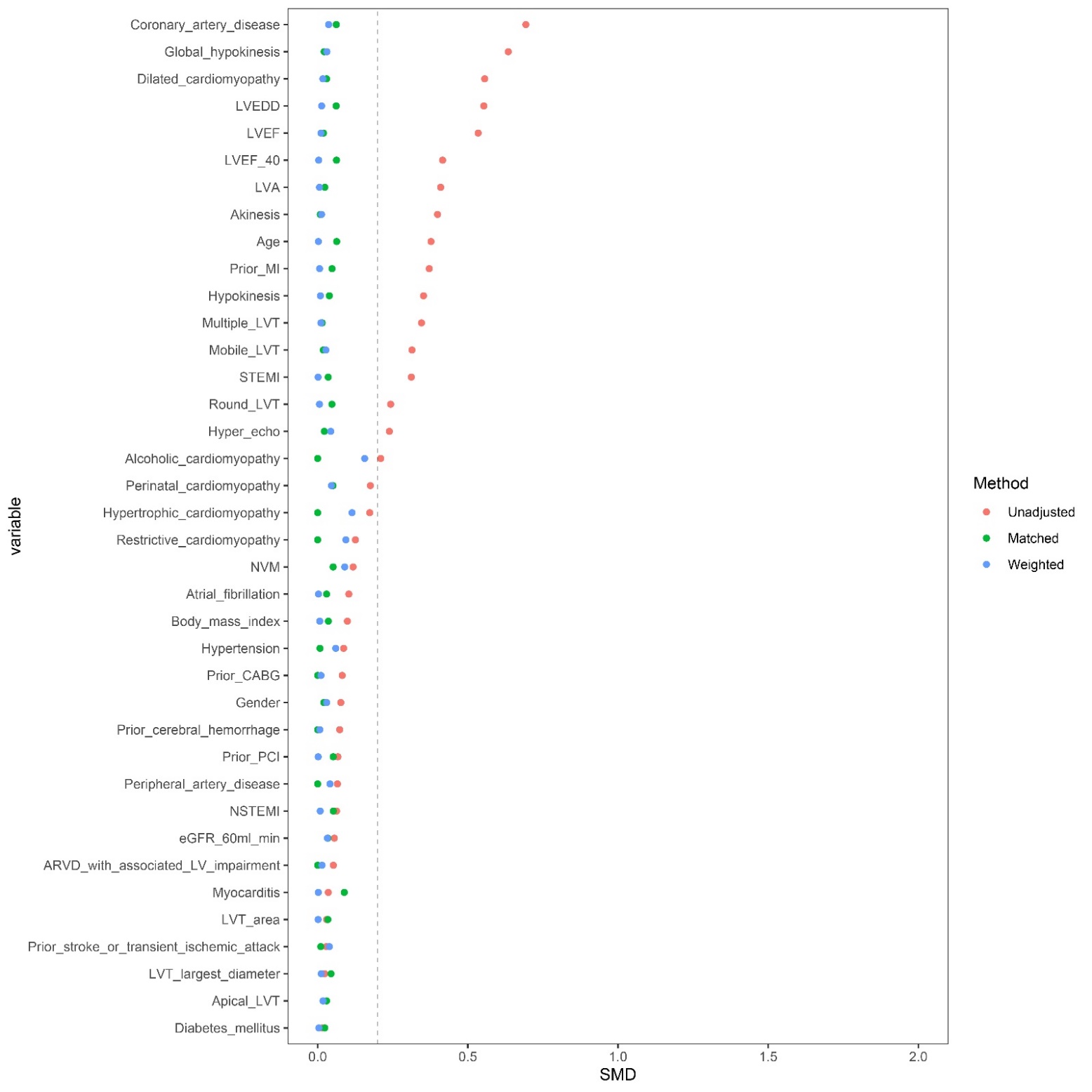


**Supplementary Fig. 3. Standardized mean difference (SMD) between the 2 groups before and after propensity score matching and propensity score weight.** eGFR=estimated glomerular filtration rate. MI=myocardial infarction. CABG=coronary artery bypass grafting. PCI=percutaneous coronary intervention. STEMI=ST-segment elevation myocardial infarction. NSTEMI=non–ST-segment elevation myocardial infarction. ARVD=arrhythmogenic right ventricular dysplasia. LV=left ventricle. NVM=noncompaction of the ventricular myocardium. LVT=left ventricular thrombus. LVEDD=left ventricular end diastolic dimension. LVEF=left ventricular ejection fraction. LVA=left ventricle aneurysms.
